# UPLC-HRMS-MS profiling of *Ludwigia adscendens* subsp. *diffusa* aerial parts and investigation of the anti-inflammatory effect

**DOI:** 10.1038/s41598-025-05183-x

**Published:** 2025-06-05

**Authors:** Enas M. Shawky, Rim Hamdy, Mohamed R. Elgindi, Mostafa H. Baky

**Affiliations:** 1 Department of Pharmacognosy, Faculty of Pharmacy, College of Pharmacy, Egyptian Russian University, Badr City, Cairo, 11829 Egypt; 2 Department of Botany and Microbiology, Faculty of Science, Cairo University, Cairo, Egypt; 3 Department of Pharmacognosy, Faculty of Pharmacy, Helwan University, Cairo, Egypt

**Keywords:** Onagraceae, *Ludwigia adscendens*, *Ludwigia stolonifera*, Metabolic profiling, Anti-inflammatory activity, Secondary metabolism, Chemistry

## Abstract

*Ludwigia adscendens* subsp. *diffusa* (Forssk.) P.H. Raven, also known as *L. stolonifera*, is an aquatic herb belonging to family Onagraceae and widely distributed in canals and drains in the Nile Delta, Egypt. The main goal of the current study is to investigate the metabolic profile of *L. adscendens* aerial parts using ultra-high performance liquid chromatography coupled with high resolution mass spectrometry (UPLC-HRMS/MS) and investigation of its anti-inflammatory activity. A total of 168 metabolites were identified by UPLC-MS/MS analysis in negative and positive modes belonging to several phytochemical classes including phenolics (57), flavonoids (26), terpenoids (25), sterols (23), fatty acids (11), coumarins (7) organic acids (5), sugar derivatives (5), lactones (4), acids (3), and glycoside (2). The UPLC-MS analysis of *L. adscendens* revealed identification of a diverse array of phytochemicals which contribute to its potential pharmacological properties. The identification of bioactive metabolites in *L. adscendens* aerial parts including gallic acid, quercetin, ellagic acid, and betulinic acid can impart biological activities including antioxidant and anti-inflammatory activities. The anti-inflammatory activity was investigated for *L. adscendens* methanol and ethyl acetate extract using nitric acid inhibition assay revealing IC_50_ of 26.4 and 23.9 µg/ml, respectively, compared to resveratrol as a standard anti-inflammatory with IC_50_ value of 14.2 µg/ml. These findings can highlight the importance of *L. adscendens* aerial parts as a potential source of bioactive metabolites.

**Supplementary Information:**

The online version contains supplementary material available at 10.1038/s41598-025-05183-x.

## Introduction

Recently, great interest has been paid to plant-based phytochemicals including phenolic acids, flavonoids, steroids, terpenoids, and others, owing to their myriad health benefits^1^. Onagraceae also known as evening primrose or willowherb family, is a family of flowering plants widely distributed in every continent, from tropical to boreal regions. Onagraceae comprises about 17 genera and 650 species of trees, shrubs, and herbs distributed into two subfamilies and seven tribes^2^. Genus *Ludwigia* is a member of the Ludwigioideae subfamily distributed in South and North America, and comprises about 82 species of aquatics plants^3^.

*Ludwigia* species are reported for their diverse biological properties including antidiabetic, cytotoxicity, antioxidant, hepatoprotective antimicrobial, and anti-inflammatory activities. For instance, *L. hyssopifolia* (G.Don) Exell. aerial parts methanolic extract showed potent anti-inflammatory activity^4^ *L. octovalvis* (Jacq.) P.H.Raven aqueous ethanolic extract showed antidiabetic effect^5^and *L. peploides* (Kunth) P.H.Raven leaves methanolic extract revealed cytotoxic, analgesic, antimicrobial, antidiarrheal and hypolipidemic properties^2^. In the Egyptian flora, *Ludwigia* genus is represented by two species: *L. erecta* (L.) Hara. and *L. stolonifera* (Guill. & Perr.) P.H.Raven^6^.

*Ludwigia adscendens* subsp. *diffusa* Forssk. Also known as *Ludwigia stolonifera*, it emerged as one of the most important aquatic plants widely distributed in canals and drains crossing the cultivated lands in the Nile Delta. *L. adscendens* is well known for its economic importance, being used in water bioremediation to help in improving drinking water quality^3^. Owing to its ability to remove toxic contaminants including heavy metals such Pb, Cd, and Cr from aquatic ecosystems, *L. stolonifera* roots and leaves are used as water biofilters^7^. Moreover, *L. stolonifera* is rich in bioactive secondary metabolites which are imparted for their biological activities including antioxidant, antidiabetic, hepatoprotective, and cytotoxic activities^3,8,9^.

Nowadays, different metabolomics tools are widely applied to profile plant-based primary and secondary metabolites^10^. LC-MS is well-suited metabolomics approach suited for the identification of non-volatile secondary metabolites in herbal materials^11^. Several studies have demonstrated that variety of antioxidant phytoconstituents also display a potent anti-inflammatory effect^12,13^. *L. adscendens* ethyl acetate extract possesses antioxidant activity^9^ so this study focuses on investigation of its anti-inflammatory effect.

The main goal of the current study is to evaluate the phytochemical profile in *L. adscendens* subsp. *diffusa* aerial parts using UPLC-MS/MS in negative and positive modes. Further investigation of the anti-inflammatory activity *L. adscendens* extract was employed using nitric oxide inhibition assay.

## Results and discussion

**Fig. 1:**
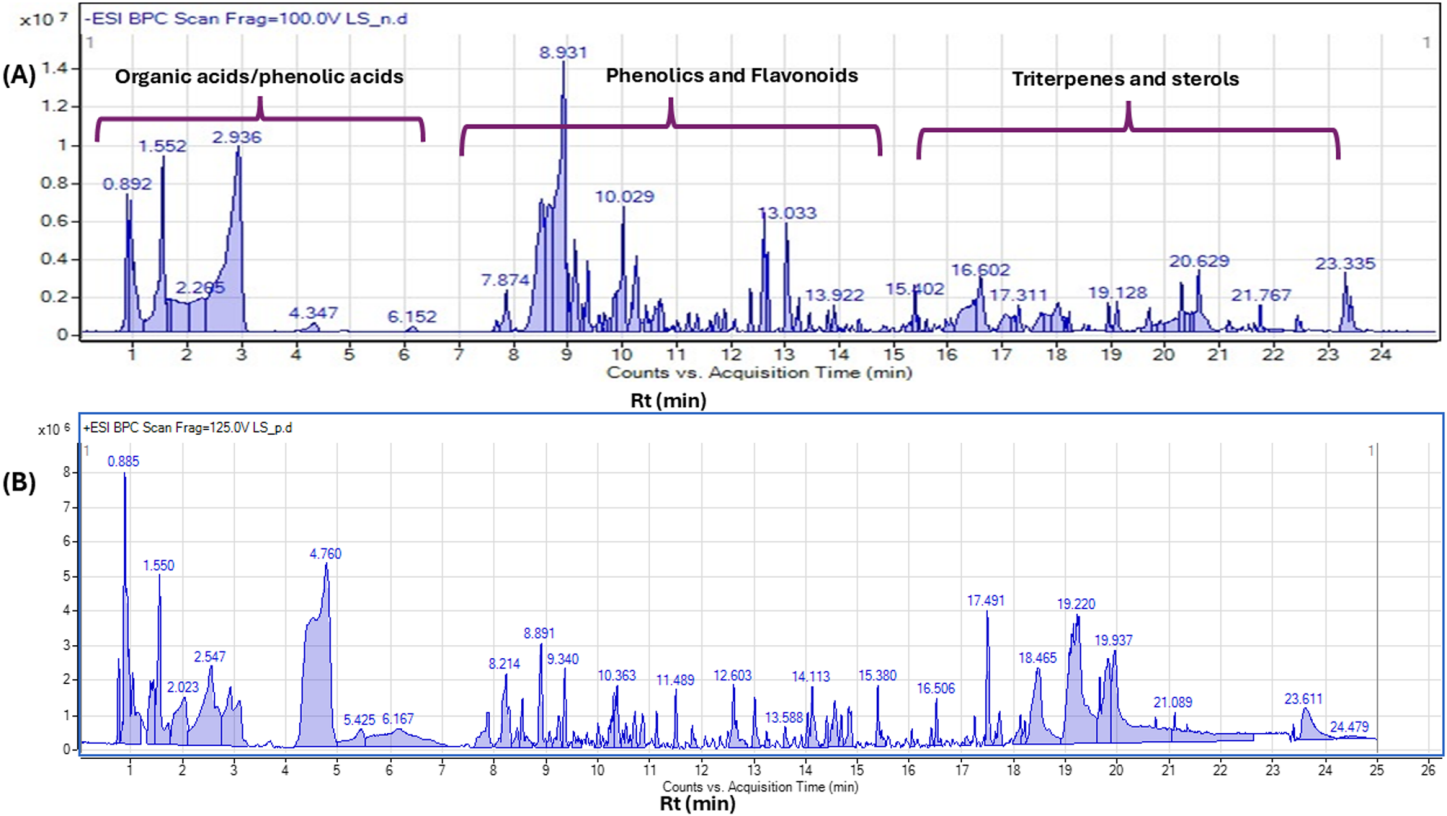
UPLC-MS base peak chromatograms of *L. adscendens* aerial parts (**A**) BPC in negative mode (**B**) BPC in positive mode.

**Fig. 2:**
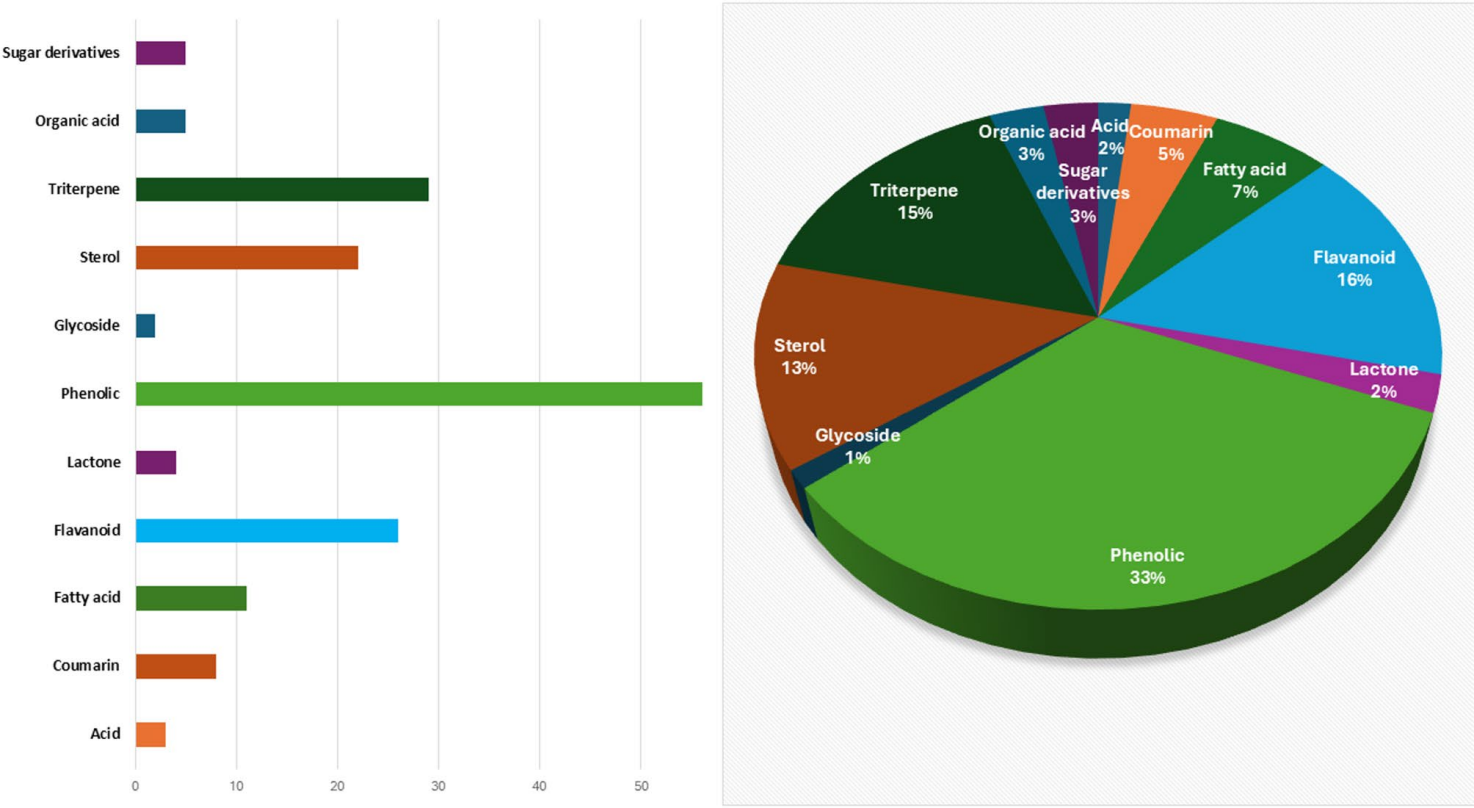
Representative bar-chart and pie chart of identified metabolites classes in *L. adscendens* aerial parts using UPLC-MS negative and positive modes.

Ultra-high performance liquid chromatography coupled with high resolution mass spectrometry (UPLC-HRMS/MS) analysis was employed for *L. adscendens* aerial parts methanol extract in both positive and negative modes (Fig. 1). Compounds were eluted within 25 min from the most polar to the least polarity ones according to the sequence of elusion. The identification was depended on comparison of the high-resolution mass spectra information with phytochemical dictionary of natural product databases and MS/MS and with that reported in the literature. A total of 168 metabolites were identified by UPLC-MS analysis in negative and positive modes (Table 1; Fig. 2).

**Table 1:** Chemical metabolites tentatively identified in *L. adscendens* aerial parts by UPLC-MS/MS at negative and positive modes.

No	Rt	Formula	Tentative identification	Class	[M-H]^−^/[M + H]^+^ m/z	Mass	Error ppm	Fragmentation	References
1	1.259	C_5_H_8_O_4_^+^	Dimethyl malonate	Acid	133.0494	132.0421	1.1	130, 70	
2	9.618	C_16_H_22_O_10_^+^	Geniposidic acid	Acid	375.1265	374.1192	4.6	282, 207, 159	^66^
3	14.43	C_15_H_20_O_4_^+^	Abscisic acid	Acid	265.1413	264.1339	4.41	243, 100	^67^
4	7.888	C_10_H_6_O_4_^+^	Coumarin-3-carboxylic acid	Coumarin	191.0337	190.0265	0.81	145, 117	
5	10.733	C_19_H_20_O_10_^+^	Khelloside	Coumarin	409.1109	408.1035	4.26	355, 248	^68^
6	11.738	C_20_H_18_O_4_^+^	Cyclocumarol	Coumarin	323.1255	322.1182	4.05	233, 184, 100	
7	12.031	C_11_H_10_O_6_^+^	Penicimarin F	Coumarin	239.0523	238.0449	4.84	209, 100	
8	12.045	C_23_H_22_O_8_^+^	6-Hydroxysumatrol	Coumarin	427.1363	426.129	4.73	351, 299,218, 100	
9	12.507	C_10_H_8_O_3_^+^	7-Methoxycoumarin	Coumarin	177.0548	176.0475	−0.93	100	
10	3.711	C_11_H_6_O_2_^+^	Ethynyl coumarin	Coumarin	171.0451	170.0379	−6.39	157, 97	
11	17.546	C_18_H_34_O_3_^−^	Ricinoleic acid	Fatty acid	297.2422	298.2495	4.23	194	
12	18.992	C_18_H_30_O_2_^−^	Linolenic Acid	Fatty acid	277.2163	278.2236	3.53	210, 159, 54	^42^
13	12.587	C_18_H_28_O_3_^+^	Colnelenic acid	Fatty acid	293.2112	292.2039	−0.35	195, 100	
14	12.987	C_12_H20O_3_^+^	Dihydrojasmonic acid	Fatty acid	213.1465	212.1392	4.63	195, 100	
15	12.997	C_18_H_30_O_3_^+^	Colneleic acid	Fatty acid	295.2268	294.2196	−0.28	195, 100	
16	13.459	C_12_H_18_O_3_^+^	Jasmonic acid	Fatty acid	211.1329	210.1256	0.08	100	
17	13.62	C_24_H_40_O_7_^+^	Ascorbyl oleate	Fatty acid	441.2822	440.275	5.5	353, 274, 203	
18	15.391	C_21_H_36_O_4_^+^	Glyceryl linolenate	Fatty acid	353.2688	352.2616	−0.6	304, 221,100	
19	15.991	C_24_H_42_O_7_^+^	Ascorbyl stearate	Fatty acid	443.2981	442.291	4.12	379, 282, 137	
20	16.244	C_18_H_28_O_2_^+^	Stearidonic acid	Fatty acid	277.2164	276.2091	3.68	235, 221, 207	^69^
21	16.845	C_18_H_30_O_2_^+^	linolenic acid	Fatty acid	279.2317	278.2244	−0.35	144, 137	^70^
22	10.359	C_28_H_24_O_16_^−^	Quercetin 3-*O*-(6’’-galloyl) -hexoside	Flavonoid	615.0963	616.1037	0.9	463, 301, 300, 271	^15^
23	11.684	C_27_H_22_O_14_^−^	Myricetin hexaacetate	Flavonoid	569.0925	570.1003	3.54	596, 553	
24	1.499	C_34_H_30_O_9_^−^	Sophoraflavanone H	Flavonoid	581.1812	582.1885	4.33	391, 341	
25	2.836	C_15_H_10_O_11_^−^	Unknown flavonoid	Flavonoid	365.0135	366.021	4.45		
26	10.229	C_21_H_20_O_12_^−^	Isoquercetin	Flavonoid	463.0862	464.0935	4.25	301, 271, 255	^29^
27	10.528	C_21_H_20_O_13_^−^	Myricetin 3-*O*-hexoside	Flavonoid	479.081	480.0883	3.91	239	^71^
28	10.538	C_20_H_18_O_11_^−^	Avicularine	Flavonoid	433.0758	434.0831	4.95	301	^72^
29	10.692	C_21_H_20_O_11_^−^	Kaempferol 3-O-hexoside	Flavonoid	447.0916	448.0988	5.34	285, 255, 227	^29^
30	10.901	C_27_H_22_O_15_^−^	Quercetin 3-(2’’-galloyl- L-hexoside)	Flavonoid	585.0855	586.093	3.56	463, 301	
31	10.951	C_20_H_18_O_10_^−^	Kaempferol 3-*O*-arabinoside	Flavonoid	417.081	418.0878	4.42	327, 285, 284	^30^
32	11.871	C_19_H_12_O_11_^−^	Quercetin succinate	Flavonoid	415.0283	416.0357	1.2	299, 255	
33	11.897	C_15_H_10_O_7_^−^	Quercetin	Flavonoid	301.0342	302.0414	4.44	178.99, 151	^30^
34	12.534	C_15_H_12_O_5_^−^	Naringenin	Flavonoid	271.06	272.0673	4.04	151, 177.019	^30^
35	15.731	C_31_H_38_O_6_^−^	Amoritin	Flavonoid	505.2548	506.262	4.45	437, 369	
36	10.286	C_21_H_18_O_13_^+^	Quercetin 3-O-glucuronide	Flavonoid	479.0822	478.0749	−0.34	303, 296	^73^
37	10.522	C_22_H_16_O_11_^+^	7-O-Galloyltaxifolin	Flavonoid	457.0741	456.0666	5.76	303, 265	
38	10.529	C_20_H_18_O_11_^+^	Quercetin-3-arabinoside	Flavonoid	435.0923	434.0851	−0.45	303, 246	^73^
39	10.554	C_15_H_10_O_7_^+^	Quercetin	Flavonoid	303.0499	302.0426	0.19	153, 181	^26^
40	10.618	C_21_H_20_O_11_^+^	Quercetin 3-deoxyhexoside	Flavonoid	449.1078	448.1007	−0.28	303, 265	^73^
41	10.652	C_13_H_14_O_9_^+^	Norbergenin	Flavonoid	315.0706	314.0632	1.97	303, 268	
42	10.912	C_20_H_18_O_10_^+^	Juglalin	Flavonoid	419.0977	418.0901	−0.18	287, 227	^73^
43	13.064	C_24_H_22_O_9_^+^	Aflavarin	Flavonoid	455.1319	454.1247	3.63	281, 195	
44	19.001	C_24_H_20_O_11_^+^	Unknown flavonoid	Flavonoid	485.1125	484.1031	1.86		
45	11.059	C_15_H_10_O_8_^−^	Myricetin	Flavonoid	317.0293	318.0364	3.91	180, 248	^74^
46	11.198	C_21_H_20_O_10_^−^	Apigenin 8-C-hexoside	Flavonoid	431.0967	432.1024	4.42	341, 311	^31^
47	13.668	C_17_H_12_O_9_^−^	Acetyl Myricetin	Flavonoid	359.0395	360.0469	3.55	316	
48	11.911	C_22_H_22_O_9_^+^	Formononetin hexoside	Glycoside	431.1315	430.1238		299, 146	
49	10.586	C_23_H_24_O_13_^−^	Syringetin-3-O-glucoside	Glycoside	507.1121	508.119	4.23	165, 299, 461	^26^
50	5.756	C_7_H_6_O_4_^+^	Patulin	Lactone	155.0316	154.0243	15.01	115	
51	5.769	C_5_H_6_O_3_^+^	Methylsuccinic anhydride	Lactone	115.0389	114.0316	0.55	101, 97, 85	
52	5.809	C_4_H_4_O_3_^+^	Succinic anhydride	Lactone	101.0232	100.0159	1.48	72, 44	
53	10.453	C_11_H_16_O_3_^+^	Loliolide	Lactone	197.1172	196.1099	0.15	179, 161	
54	0.987	C_7_H_10_O_7_^−^	Homocitric acid	Organic acid	205.0347	206.0419	2.9	111	^75^
55	1.1	C_6_H_8_O_7_^−^	Citric acid	Organic acid	191.0192	192.0264	2.47	111, 87, 76, 57	^42^
56	1.115	C_4_H_6_O_5_^−^	Malic acid	Organic acid	133.0139	134.0212	3.64	115, 71	^42^
57	1.722	C_5_H_8_O_5_^−^	Citramalic acid	Organic acid	147.0294	148.0366	4.19	113	
58	4.372	C_6_H_6_O_6_^−^	Dehydroascorbic acid	Organic acid	173.0084	174.0157	3.61	155	
59	11.337	C_10_H_10_O_4_^−^	Ferulic acid	Phenolic	193.0498	194.0571	4.2	149	^11^
60	1.028	C_7_H_6_O_5_^−^	Gallic acid	Phenolic	169.014	170.0212	4.2	125, 124	^15^
61	1.794	C_13_H_16_O_10_^−^	Gallic acid hexoside	Phenolic	331.0656	332.0728	3.95	313, 169, 168, 125	^16^
62	2.905	C_6_H_6_O_3_^−^	Pyrogallol	Phenolic	125.024	126.0314	1.72	97, 81	^76^
63	4.069	C_14_H_16_O_10_^−^	4-O-Galloylquinic acid	Phenolic	343.0658	344.0729	4.59	183, 161	^77^
64	4.075	C_20_H_20_O_14_^−^	2,6-Digalloylglucose	Phenolic	483.0757	484.0829	2.26	439, 331, 313, 271, 211, 169, 125.	^15^
65	7.741	C_14_H_10_O_10_^−^	Ellagic acid dihydrate	Phenolic	337.0187	338.026	4.12	297, 269	
66	8.138	C_13_H_10_O_8_^−^	Pyrogallol gallate	Phenolic	293.0289	294.0363	4.94	179, 151	
67	8.268	C_8_H_8_O_5_^−^	Methyl gallate	Phenolic	183.0292	184.0365	4.23	167, 124	^18^
68	8.388	C_12_H_14_O_8_^−^	Dihydroxybenzoic acid pentoside	Phenolic	285.0603	286.0676	4.46	153, 152, 109, 108	^16^
69	8.529	C_16_H_16_O_10_^−^	Feruloylisocitric acid	Phenolic	367.0657	368.0729	3.84	163, 103	
70	8.713	C_9_H_8_O_6_^−^	Dihydroxycaffeic acid	Phenolic	211.0239	212.0302	3.89	179, 153	
71	9.061	C_15_H_12_O_10_^−^	Methylenedigallic acid	Phenolic	351.0342	352.0415	8.74	263, 215, 169	
72	9.226	C_15_H_14_O_7_^−^	Gallocatechin	Phenolic	305.069	306.0758	4.3	261, 125	
73	10.246	C_14_H_6_O_8_^−^	Ellagic acid	Phenolic	300.9983	302.0055	−5.9	253, 258	^20^
74	10.283	C_28_H_12_O_16_^−^	Gallagic acid	Phenolic	603.0025	604.0098	2.53	573, 483	
75	10.797	C_14_H_10_O_9_^−^	Digallic acid	Phenolic	321.0239	322.0313	4.6	169, 125	^76^
76	10.996	C_21_H_14_O_12_^−^	Flavogallonic acid	Phenolic	457.0396	458.047	3.82	375, 169	
77	11.185	C_15_H_8_O_8_^−^	3-O-methylellagic acid	Phenolic	315.0132	316.0205	3.31	299, 216	^76^
78	11.445	C_28_H_24_O_15_^−^	3’’-Galloylquercitrin	Phenolic	599.1017	600.109	4.42	463, 301, 300, 169, 125, 98	^15^
79	11.61	C_14_H_12_O_8_^−^	Fulvic acid	Phenolic	307.044	308.0509	4.18	191, 173	
80	12.222	C_16_H_10_O_8_^−^	3,3’-Di-O-methylellagic acid	Phenolic	329.0288	330.036	4.53	314, 285	^76^
81	12.298	C_10_H_10_O_3_^−^	4-methoxycinnamic acid	Phenolic	177.0549	178.0616	4.88	163, 149	^78^
82	14.576	C_17_H_26_O_4_^−^	Gingerol	Phenolic	293.1746	294.1819	4.75	277, 221, 177	^24^
	3.864	C_22_H_18_O_14_^+^	Dimethylellagic acid glucuronide	Phenolic	507.0748	506.0673	5.32	467, 328, 171	
84	3.977	C_9_H_10_O_5_^+^	Syringic acid	Phenolic	199.0576	198.0502	−0.77	141, 97	
85	4.068	C_14_H_16_O_10_^+^	4-*O*-Galloylquinic acid	Phenolic	345.0814	344.0746	0.99	328, 199, 141	^76^
86	4.862	C_6_H_6_O_3_^+^	Pyrogallol	Phenolic	127.0388	126.0316	5.09	107, 79	
87	5.259	C_9_H_10_O_6_^+^	2-Hydroxyethyl gallate	Phenolic	215.0525	214.0445	6.11	176, 141	
88	8.386	C_20_H_16_O_13_^+^	Ellagic acid hexoside	Phenolic	465.0665	464.0595	0.03	299	
89	8.441	C_14_H_6_O_8_^+^	Ellagic acid	Phenolic	303.0135	302.0063	0.45	285, 267, 230, 160	^20^
90	8.455	C_13_H_8_O_7_^+^	Urolithin M5	Phenolic	277.0342	276.0269	6.48	238, 200	
91	9.08	C_19_H_22_O_9_^+^	Aloesin	Phenolic	395.1312	394.1238	0.15	319, 217, 193	
92	9.145	C_13_H_8_O_8_^+^	Brevifolincarboxylic acid	Phenolic	293.0292	292.0219	−0.28	243, 194	
93	9.183	C_13_H_10_O_8_^+^	Pyrogallol gallate	Phenolic	295.0449	294.0377	6.19	238, 153	
94	9.465	C_20_H_26_O_9_^+^	Butyl chlorogenat	Phenolic	411.1625	410.1551	5.43	287, 189	
95	9.512	C_10_H_10_O_7_^+^	3-Galloyl glycerinaldehyde	Phenolic	243.0477	242.0404	4.59	218, 169	
96	9.58	C_21_H_26_O_10_^+^	Fortuneanoside B	Phenolic	439.1579	438.1506	−0.09	409, 207	
97	10.562	C_21_H_16_O_13_^+^	Ellagic acid acetyl-pentoside	Phenolic	477.0663	476.0591	5.51	435, 303, 252	
98	10.642	C_11_H_12_O_4_^+^	Sinapyl aldehyde	Phenolic	209.0782	208.0712	5.85	104, 191	^26^
99	10.716	C_19_H_16_O_11_^+^	Urolithin C 3-glucuronide	Phenolic	421.074	420.0668	4.7	227, 199	
100	11.155	C_11_H_12_O_6_^+^	Lignicol	Phenolic	241.0683	240.0611	0.27	227, 187	
101	11.192	C_29_H_34_O_16_^+^	Ombuoside	Phenolic	639.1918	638.1845	5.36	587, 386, 296	
102	11.296	C_11_H_14_O_3_^+^	Zingerone	Phenolic	195.0991	194.0917	5.45	144, 100	
103	11.411	C_19_H_22_O_8_^+^	Oleuropein aglycone	Phenolic	379.1367	378.1294	5.65	294, 207, 100	
104	11.444	C_14_H_18_O_5_^+^	Hypoxymarin C	Phenolic	267.1199	266.1126	5.87	207, 144, 100	
105	11.588	C_11_H_14_O_5_^+^	Butyl gallate	Phenolic	227.0889	226.0817	6.38	171, 125, 100	
106	11.611	C_18_H_12_O_10_^+^	Repenol	Phenolic	389.0482	388.0406	5.1	301, 233,	
107	11.659	C_13_H_14_O_7_^+^	Feruloyl Lactate	Phenolic	283.0791	282.072	5.56	223, 184, 144, 100	
108	12.282	C_14_H_16_O_4_^+^	Prenyl caffeate	Phenolic	249.1098	248.1025	6.13	226, 100	
109	12.44	C_17_H_26_O_3_^+^	Paradol	Phenolic	279.1929	278.1854	6.06	137, 100, 68	
110	12.456	C_22_H_20_O_9_^+^	Hesperetin Triacetate	Phenolic	429.1155	428.1081	0.25	381, 320, 246	
111	13.908	C_13_H_10_O_6_^+^	Maclurin	Phenolic	263.0562	262.0477	0.02	223, 100	
112	14.59	C_17_H_10_O_9_^+^	Distemonanthin	Phenolic	359.0398	358.0325	6.82	309, 227, 100	
113	8.923	C_15_H_14_O_10_^−^	2-O-caffeoyl glucarate	Phenolic	353.0502	354.0574	4.7	191, 179	^79^
114	2.585	C_15_H_14_O_10_^+^	2-Caffeoylisocitric acid	Phenolic	355.0636	354.0563	2.83	294, 144, 112	
115	10.939	C_21_H_14_O_12_^+^	Tergalloyl	Phenolic	459.0554	458.0482	5.1	310, 209, 185	
116	12.61	C_37_H_60_O_12_^−^	Momordicoside E	Sterol	695.3989	696.4063	4.96	421	^80^
117	12.168	C_21_H_34_O_6_^+^	Sarcostin	Sterol	383.2404	382.2333	5.95	355, 249, 100	
118	12.358	C_21_H_32_O_6_^+^	Marsdenin	Sterol	381.2247	380.2174	6.57	320, 188,100	
119	14.277	C_38_H_56_O_9_^+^	Bruceajavanone C	Sterol	657.3973	656.3905	2.95	365, 316, 237, 100	
120	14.878	C_32_H_46_O_5_^+^	Ganoderic acid T-Q	Sterol	511.3399	510.3327	2.99	453, 365, 239, 100	
121	15.659	C_23_H_34_O_6_^+^	Bipindogenin	Sterol	407.2409	406.2333	3.66	379, 345, 100	
122	16.555	C_39_H_54_O_7_^+^	2-O-caffeoyl maslinic acid	Sterol	635.3937	634.3865	−4.35	531, 383, 347	^81^
123	18.117	C_37_H_62_O_11_^+^	Cyclopassifloside II	Sterol	683.4341	682.4267	−0.11	639, 527, 469,277, 263, 104	
124	19.399	C_30_H_50_O_9_^+^	Notoginsenoside R10	Sterol	555.3507	554.3434	−0.27	487, 443, 365, 256, 137	
125	21.279	C_42_H_62_O_6_^+^	28-O-benzyl-3-O-glutaryl-dihydrobetulinic acid	Sterol	663.4658	662.4584	5.58	616, 575, 531, 487	
126	21.518	C_41_H_62_O_6_^+^	β-Sitosterol-3-O-arabinobenzoate	Sterol	651.4591	650.4519	4.95	575, 531, 487	
127	21.638	C_41_H_60_O_6_^+^	3β-trans-Sinapoyloxylup-20(29)-en-28-ol	Sterol	649.4495	648.4422	4.3	575, 487, 443	
128	22.603	C_39_H_56_O_4_^+^	Stigmasterylferulate	Sterol	589.4283	588.421	3.88	575, 487, 394	
129	23.515	C_29_H_48_^+^	Stigmastadiene	Sterol	397.3827	396.3754	4.34	335, 144, 68	
130	13.556	C_35_H_54_O_16_^+^	Sarmentologenin	Sterol	731.346	730.339	4.18	353, 203, 100	
131	14.845	C_31_H_50_O_7_^−^	Unknown sterol	Sterol	533.3454	534.3528	3.19		
132	15.206	C_30_H_42_O_11_^−^	Hellebrigenin hexoside	Sterol	577.2661	578.2732	4.31	415	^82^
133	15.596	C_29_H_44_O_5_^−^	Camphoratin B	Sterol	471.3099	472.3171	−0.76	453, 413	^83^
134	16.25	C_28_H_32_O_4_^−^	Sterostrein A	Sterol	431.2186	432.2259	3.67	413, 387	
135	18.206	C_29_H_46_O_8_^−^	Viticosterone E	Sterol	521.3095	522.3169	4.71	503, 477	
136	21.081	C_37_H_56_O_8_^−^	Palustrisolide E	Sterol	627.387	628.3943	4.52	609, 583	
137	13.273	C_25_H_34_O_8_^−^	unknown sterol	Sterol	461.2161	462.2234	4.14		
138	1.115	C_6_H_12_O_6_^−^	Glucose	Sugar	179.0558	180.063	2.17	101, 89	^76^
139	0.948	C_4_H_8_O_5_^−^	L-threonic acid	Sugar derivatives	135.0295	136.0367	3.27	101, 69	
140	1.367	C_8_H_16_O_8_^−^	Glucitol acetate	Sugar derivatives	239.0763	240.0836	3.94	229, 211	
141	1.603	C_6_H_10_O_6_^−^	Gluconolactone	Sugar derivatives	177.04	178.0472	2.85	159, 141	
142	2.036	C_6_H_10_O_5_^−^	Meglutol	Sugar derivatives	161.0449	162.0522	3.69	159, 141, 123	
143	12.484	C_30_H_48_O_6_^−^	Protobassic acid	Triterpene	503.3355	504.3427	4.66	458, 458	^84^
144	12.967	C_15_H_22_O_4_^+^	Deoxyartemisinin	Triterpene	267.1568	266.1495	6.67	213, 100	
145	13.304	C_34_H_36_O_11_^+^	Orbiculin F	Triterpene	621.2308	620.2234	3.76	381, 227	
146	14.366	C_30_H_46_O_5_^−^	Bassic acid	Triterpene	485.325	486.3323	4.66	487, 451	
147	14.411	C_31_H_48_O_7_^−^	Phytolaccagenin	Triterpene	531.3298	532.3372	4.61	509, 473	
148	14.455	C_30_H_48_O_5_^−^	Asiatic Acid	Triterpene	487.3407	488.3482	5.19	453, 417	^85^
149	14.459	C_31_H_48_O_8_^−^	Quadrangularic acid F	Triterpene	547.325	548.333	3.94	509, 473	
150	14.661	C_29_H_46_O_5_^−^	Stoloniferone S	Sterol	473.3253	474.3326	3.41	455, 419	^86^
151	15.875	C_39_H_54_O_7_^−^	2-O-caffeoyl maslinic acid	Triterpene	633.3767	634.384	3.98	615, 579, 561	
152	16.483	C_28_H_44_O_11_^−^	Forskoditerpenoside C	Triterpene	555.2824	556.2894	4.72	521, 485	
153	16.606	C_31_H_44_O_13_^−^	Ligurobustoside I	Triterpene	623.2688	624.2758	−1.86	549, 513, 495	
154	16.927	C_30_H_48_O_4_^−^	Hederagenin	Triterpene	471.3461	472.3535	3.88	453, 417	^87^
155	16.927	C_30_H_48_O_4_^−^	Maslinic Acid	Triterpene	471.3461	472.3535	3.64	423, 393, 405	^37^
156	17.69	C_30_H_46_O_4_^−^	Glycyrrhetic acid	Triterpene	469.3304	470.339	4.67	425, 407	^88^
157	18.673	C_40_H_56_O_7_^−^	Uncarinic acid B	Triterpene	647.3927	648.3997	3.86	613, 577, 559	^89^
158	19.194	C_30_H_48_O_3_^−^	Betulinic acid	Triterpene	455.3513	456.3585	4.55	327, 317, 353,409, 437	^35^
159	22.582	C_36_H_54_O_7_^−^	Integracide H	Triterpene	597.3767	598.3841	3.99	571, 535, 517	
160	22.669	C_36_H_60_O_8_^−^	Fasciculic acid A	Triterpene	619.4185	620.4257	4.72	581, 545, 527	
161	13.173	C_24_H_38_O_7_^+^	Bisacremine F	Triterpene	439.2666	438.2594	5.28	381, 311, 100	
162	14.226	C_30_H_44_O_4_^+^	3-oxoglycyrrhetinic acid	Triterpene	469.3313	468.3241	4.37	409, 365, 335, 207, 100	
163	16.884	C_30_H_46_O_3_^+^	Mangiferonic acid	Triterpene	455.352	454.3442	−0.32	437, 383, 318, 207, 137	^90^
164	16.903	C_30_H_44_O_2_^+^	Ganoderal A	Triterpene	437.3419	436.3344	4.36	383, 318, 207	
165	12.358	C_30_H_42_O_3_^+^	Dysolenticin B	Triterpene	451.3207	450.3134	1.17	249	
166	12.665	C_30_H_44_O_3_^+^	Ganoderic acid SZ	Triterpene	453.3365	452.3293	−0.02	351, 262	
167	12.691	C_36_H_54_O_8_^+^	Asiatic acid triacetate	Triterpene	615.3892	614.382	−0.64	453, 351	
168	12.788	C_21_H_36_O_6_^+^	Dihydrosarcostin	Triterpene	385.256	384.2486	−0.26	281, 100	

## Chemical metabolites identified in *Ludwigia stolonifera* by UPLC-MS/MS negative and positive modes

UPLC-MS/MS analysis of *L. adscendens* aerial parts in negative and positive (Fig. 1A and B) revealed the annotation of 168 metabolites (Table 1; Fig. 2) belonging to several phytochemical classes including phenolics (57), flavonoids (26), terpenoids (25), sterols (22), fatty acids (11), coumarins (8) organic acids (5), sugar derivatives (5), lactones (4), acids (3), and glycoside (2). Flavonoids and phenolics were identified as the most abundant metabolite classes which enhance the biological properties of L. *adscendens* including antioxidant and anti-inflammatory effects.

### Phenolic compounds

Phenolic compounds were identified as the most abundant class represented by 57 peaks. Phenolic compounds are ubiquitously distributed phytochemicals found in most plants and possess numerous bioactive properties including antioxidant, antimicrobial, and anti-inflammatory^14^. Peaks 60, 61, and 75 were assigned to gallic acid (*m/z* 169.0140, C_7_H_6_O_5_^−^)^15^ and its derivatives, gallic acid hexoside (C_13_H_16_O_10_^−^)^16^ and digallic acid (C_14_H_10_O_9_^−^), respectively. Gallic acid and its derivatives are potential biological importance including antioxidant, anti-inflammatory, and antimicrobial properties^17^. Methyl gallate (peak 67) and pyrogallol gallate (peak 66) appeared at *m/z* [M-H]^−^ 183.0292 (C_8_H_8_O_5_^−^) and 293.0289 (C_13_H_10_O_8_^−^)^18^respectively. Gallate deratives were previously identified in *L. adscendens* aerial parts^3^ and has been reported to exhibit hepatoprotective and anticancer effects^19^. Peaks 73, 74, and 18 were annotated for ellagic acid (*m/z* [M-H]^−^ 300.9983 C_14_H_6_O_8_)^20^ and its derivatives, such as gallagic acid and ellagic acid dihydrates, respectively. Ellagic acid and its derivatives are ellagitannins with potent antioxidant and antitumor properties^21^. Ferulic acid (peak 59) was identified (*m/z* [M-H]^−^ 193.0498 C_10_H_10_O_4_^−^) is a hydroxycinnamic acid with antioxidant and anti-inflammatory properties^22^. Peak 70 were assigned to dihydroxycaffeic acid (*m/z* [M-H]^−^ 211.0239 C_9_H_8_O_6_^−^) which possesses strong neuroprotective and anti-inflammatory properties^23^. Gingerol (Peak 59) was detected in *L. adscendens* aerial parts and is well known for its anti-inflammatory and antioxidant activities^24^. Simple phenolics were identified including pyrogallol (peak 86) [M + H]^+^ at *m/z* 127.0388 (C_6_H_6_O_3_^+^) and 2-hydroxyethyl gallate (peak 87) [M + H]^+^ at *m/z* 215.0525 (C_9_H_10_O_6_^+^) with antioxidant and anti-inflammatory properties^25^. Peak 107 was identified as feruloyl lactate and peak 98 was identified as Sinapyl aldehyde [M + H]^+^ at *m/z* 209.0782 (C_11_H_12_O_4_^+^)^26^. Sinapyl aldehyde is a precursor in lignin biosynthesis and contributes to plant cell wall integrity and well known for its antioxidant and anti-inflammatory properties^26^. Peak 102 was assigned to zingerone, which is a phenolic compound with anti-inflammatory and antioxidant properties^27^.

### Flavonoids

Flavonoids, a group of natural substances with variable phenolic structures with potential health benefits owing to their anti-oxidative, anti-inflammatory, anti-mutagenic and anti-carcinogenic properties^28^. Flavonoids represented by 26 peaks were identified in *L. adscendens* aerial parts. Both *O-* & *C*-flavonoid glycosides were identified by different fragmentation pattern that distinguished between the two types of glycosidic linkages. Flavonols and flavone *O*-glycosides were identified according to neutral loss of sugar moieties; [M*-*H]^−^ [179, 161, 149 & 131 *amu*] which assigned for ions loss of (hexose, deoxyhexose, and pentose units), respectively. Peaks 33 and 26 were annotated as quercetin (*m/z* [M-H]^−^ 301.0342 C_15_H_10_O_7_^−^) and quercetin 3-*O*-hexoside (*m/z* 463.0862 C_21_H_20_O_12_^−^), respectively. Peaks 29, 31 and 46 were assigned to kaempferol-3-*O*-hexoside^29^ kaempferol-3-*O*-arabinoside^30^ and apigenin-8-C-hexoside^31^ respectively. Moreover, myricetin (peak 45) (*m/z* 317.0293 C_15_H_10_O_8_^−^) and myricetin-3-O-hexoside (peak 27) (*m/z* 479.0810 C_21_H_20_O_13_^−^) were identified in *L. adscendens* aerial parts. Peak 30 and peak 22 were annotated as quercetin 3-(2’’-galloyl-pentoside) (*m/z* [M-H]^−^ 585.055 C_27_H_22_O_15_) and quercetin 3-O-(6’’-galloyl-hexoside) (*m/z* [M-H]^−^ 615.0963 C_28_H_42_O_16_^−^) which is related to previously isolated compounds from in *L. adscendens* aerial parts^3^. Moreover, peaks 36, 38, and 40 were identified as quercetin derivatives^26^ and including quercetin 3-*O*-glucuronide [M + H]^+^ at *m/z* 479.0822 (C_21_H_18_O_13_^+^), quercetin 3-*O*-pentoside [M + H]^+^ at *m/z* 435.0923 (C_20_H_18_O_11_^+^), and quercetin 3-*O*-deoxyhexoside [M + H]^+^ at *m/z* 449.1078 (C_21_H_16_O_13_^+^)^32^ respectively. Quercetin derivatives are reported for their antioxidant and anti-inflammatory properties^33^. Quercetin has been reported to modulate signaling pathways involved in cancer progression^32^. Flavonoids can contribute to the biological importance of *L. adscendens* aerial parts owing to their myriad pharmacological properties.

### Triterpenes and sterols

Triterpenoids represented by 25 peaks were identified in *L. adscendens* aerial parts. Triterpenoids play a pivotal role in human health owing to their pharmacological activities including antidiabetic properties, neuropharmacological, and anti-inflammatory effects^34^. Peaks 143, 148, and 160 were assigned to protobassic acid (*m/z* [M-H]^−^ 503.3355 C_30_H_48_O_6_^−^), asiatic acid (*m/z* [M-H]^−^ 487.3407 C_30_H_48_O_5_^−^), and betulinic acid (*m/z* [M-H]^−^ 455.3513 C_30_H_48_O_3_^−^)^35^. Such triterpenoids were reported for their significant biological activities such as anti-inflammatory, anticancer, and hepatoprotective effects^36^. Peak 155 was annotated as maslinic acid (*m/z* [M-H]^−^ 471.3461 C_30_H_48_O_4_^−^) which a pentacyclic triterpenoid with antioxidant and anti-inflammatory properties^37^. Hederagenin (peak 154) was previously identified as the aglycone of triterpenoid saponins isolated from *L. adscendens* aerial parts^3^. It has been reported for its potential pharmacological activities including antitumor, anti-inflammatory, antidepressant, antineurodegenerative, antihyperlipidemic, antidiabetic, and antiviral activities^38^. Moreover, triterpenoids were detected in positive mode among which peak 169, 170, and 171 were identified as ganoderic acid, asiatic acid triacetate and dihydrosarcostin, respectively. These triterpenoid compounds are found in medicinal plants and contribute to their antioxidant and anti-inflammatory properties^36^.

Likewise, sterols were identified by 23 peaks represented mainly by stoloniferone S (Peak 150) and viticosterone E (Peak 135) with potential bioactive properties^39^. Plant sterols play a pivotal role in human health through several biological properties including cardioprotective, neuroprotective, and anti-aging^40^. Moreover, peak 116 and 131 were assigned to momordicoside E (C_37_H_60_O_12_) and agosterol F (C_31_H_50_O_7_) which are steroidal saponin with anti-inflammatory properties^41^. Moreover, sterols were detected in positive mode represented by several peaks among which 117, 126, 128, and 129 were identified as sarcostin [M + H]^+^ at *m/z* 383.2404 (C_21_H_34_O_6_^+^), *β*-sitosterol-3-*O*-arabinobenzoate [M + H]^+^ at *m/z* 651.4591 (C_41_H_62_O_6_^+^), Stigmasterylferulate [M + H]^+^ at *m/z* 589.4283 (C_39_H_56_O_4_^+^), and stigmastadiene [M + H]^+^ at *m/z* 397.3827 (C_29_H_48_^+^).

### Fatty acids

Fatty acids were detected mainly by 11 peaks, among which linolenic acid (peak 12)^42^ and ricinoleic acid (peak 13) were identified in negative mode. Linolenic acid [M-H]^−^ at *m/z* 277.2163 (C_18_H_30_O_2_^−^) is an essential omega-3 fatty acid with anti-inflammatory and cardioprotective effects^43^. Moreover, ricinoleic acid [M-H]^−^ at *m/z* 297.2422 (C_18_H_34_O_3_^−^) is a hydroxy fatty acid with antimicrobial and anti-inflammatory properties^44^. Peaks 15 and 20 were assigned to colneleic acid [M + H]^+^ at *m/z* 295.2268 (C_18_H_30_O_3_^+^)^45^ and stearidonic acid [M + H]^+^ at *m/z* 277.2164 (C_18_H_28_O_2_^+^)^10,11^.

### Coumarins

Coumarins represented by 7 peaks were detected in *L. adscendens* aerial parts and detected only in positive mode. Coumarins are considered as biologically active metabolites with potential pharmacological properties including anticoagulant, anti-inflammatory, and anticancer^46^. Peaks 10, 4, 7, and 9 were identified as ethynyl coumarin [M + H]^+^ at *m/z* 171.0451 (C_11_H_6_O_2_^+^), coumarin-3-carboxylic acid, penicimarin F, and 7-methoxycoumarin [M + H]^+^ at *m/z* 419.0977 (C_20_H_18_O_10_^+^).

### Organic acids

Aliphatic organic acids are the important bioactive compounds found in medicinal plants and play a key role in flavor, maintain nutritional value as well as their characteristic taste^47,48^. Among organic acids, malic and citric acids are mainly produced in the tricarboxylic acid cycle and accumulated in various plant species^47^. Peaks 55 and 56 were assigned to citric acid (*m/z* [M-H]^−^ 191.0192 C_6_H_8_O_7_) and malic acid (*m/z* [M-H]^−^ 133.0139 C_4_H_6_O_5_)^42^. These acids also contribute to the sour taste of plant tissues and play roles in pH regulation^48^. Peak 58 was assigned to dehydroascorbic acid *m/z* 173 C_6_H_6_O_6_^−^^49^ indicating the presence of ascorbic acid metabolism in *L. adscendens* aerial parts which imparts to its potent antioxidant potential.

## Anti-inflammatory activity

**Fig. 3:**
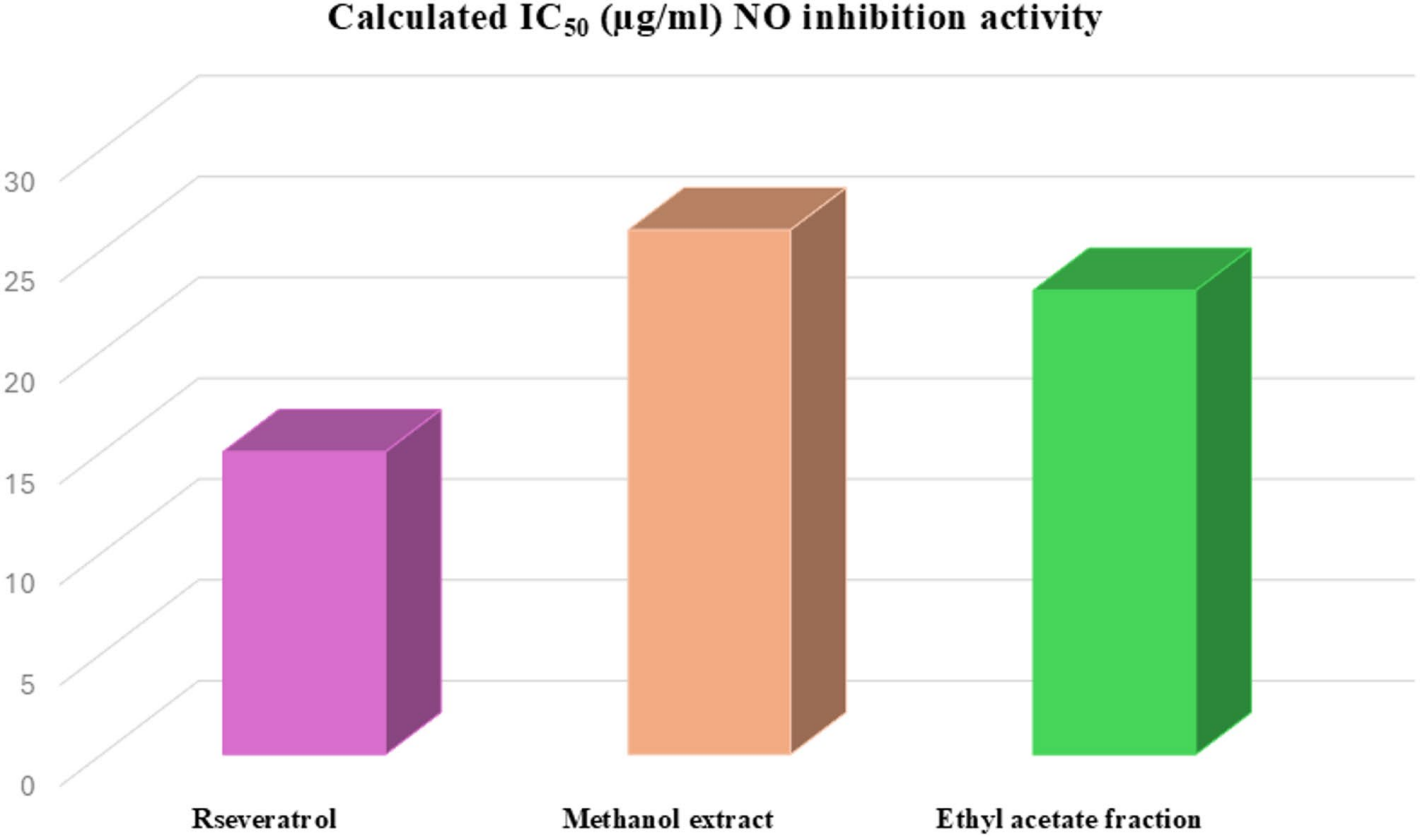
Calculated IC_50_ (µg/ml) of *L. adscendens* aerial parts by NO inhibitory activity assay.

In daily routine, the human body is largely exposed to inflammation by environmental pollutants, infections (bacteria, viruses, and fungi) and other physical and chemical agents^50^. Inflammation is a protective strategy that stimulate immune response to protect from tissue injury and other noxious conditions and promote the healing of damaged tissue^50^. Recently, several diseases were linked to the inflammatory response including atherosclerosis, Alzheimer’s disease, cancer, and cardiovascular diseases^13^. Elevation of NO level is used as a marker for inflammatory response as manifested by elevated exhaled nitric oxide (NO) in asthmatics which indicate airways inflammation^13,51^. NO is an important chemical mediator produced by endothelial cells, macrophages, and neurons and play a key role in the immune system’s host defense mechanism and regulate blood vessel tone in vascular systems^52^. NO is considered as a pro-inflammatory mediator that induces inflammation due to over production in abnormal situations^13,53^. Hence, inhibition of excess nitric oxide is one of the possible mechanisms of anti-inflammatory agents. Recently, plant-based phytochemicals identified in medicinal plants’ crude extracts and/or pure compounds, are widely used as potential sources of anti-inflammatory agents^54^. Several phytoconstituents widely distributed in plants and possess anti-inflammatory activity including phenolics, flavonoids, terpenoids, steroids, and saponins^54,55^. The ant-inflammatory activity of *L. adscendens* aerial parts methanol extract and ethyl acetate fraction was investigated via NO inhibitory assay (Table S1). Results revealed that methanol extract and ethyl acetate fraction showed potent anti-inflammatory with calculated IC_50_ of 26.4 and 23.9 µg/ml, respectively, compared to resveratrol as standard anti-inflammatory with IC_50_ value of 14.2 µg/ml (Fig. 3). Such anti-inflammatory activity of *L. adscendens* aerial parts is manifested by its richness with bioactive phytochemicals including phenolics, flavonoids, triterpenoids, and steroids. Several metabolites were identified and reported for their antioxidant and anti-inflammatory activities. Gallic acid and its derivatives are potential biological importance including antioxidant, anti-inflammatory, and antimicrobial properties^17^. Gallic acid can reduce inflammation via inhibition of proinflammatory cytokines^56^. Moreover, quercetin^57^ ellagic acid^58^ and betulinic acid^59^ contributes to the significant antioxidant, and anti-inflammatory activity^33^ of *L. adscendens* aerial parts. Such results are compatible with other previous studies on several species belonging to the family Onagraceae, which reported to exert a potent anti-inflammatory activity^60^. In another study, *Jussiaea repens* L. aerial parts ethylacetate extract revealed in vitro anti-inflammatory activity^61^. *Epilobium angustifolium* and *Epilobium montanum* aerial parts dichloromethane extracts were tested for the anti-inflammatory activity revealing a potent effect^62^.

## Conclusion

Phytochemical profiling of *L. adscendens* aerial parts via UPLC-MS/MS analysis in negative and positive modes was introduced herein. A total of 168 metabolites were identified belonging to several phytochemical classes including phenolics, flavonoids, terpenoids, sterols, fatty acids, coumarins, organic acids, sugar derivatives, lactones, acids, and glycoside. Several metabolites were identified in *L. adscendens* aerial parts with significant biological importance including gallic acid and gallate derivatives, quercetin derivatives, ellagic acid, gingerol and betulinic acid which can contribute to the antioxidant and anti-inflammatory activities. Investigation of the anti-inflammatory activity of *L. adscendens* methanol and ethyl acetate extract via nitric acid inhibition assay revealed potent activity with IC_50_ of 26.4 and 23.9 µg/ml, respectively, compared to resveratrol with IC_50_ value of 14.2 µg/ml. These findings can highlight the importance of *L. adscendens* aerial parts as a potential source of bioactive metabolites. Further isolation and biological investigation of the bioactive metabolites using different chromatographic techniques are recommended in future work.

## Materials and methods

### Plant material

The aerial parts of *L. adscendens* subsp. *diffusa* (Forssk.) P.H. Raven were collected from the Nile River at El Qanatir Al-Khayriyah, El Qulyoubia governorate (30.193583°N 31.132064°E), Egypt in September 2024. The botanical identification of the plant was confirmed by Prof. Dr. Rim Hamdy, Professor of plant taxonomy, Botany Department, Faculty of Science, Cairo University, Egypt. A voucher specimen with the number Lus2/2024, has been deposited at the Pharmacy Department of the Faculty of Pharmacy, Egyptian Russian University.

### Plant extraction

The air-dried aerial parts of *L. adscendens* (250 g) were macerated in methanol at room temperature, stirring occasionally, and the operation was repeated three times until being exhausted. The dried methanol extract was obtained by concentrating under reduced pressure using a Rotary evaporator at 50 ^o^C to yield 30 g dry methanol extract. About 10 g was kept in closed contained and kept in the refrigerator for UPLC-MS analysis. About 20 g of the obtained methanol extract was suspended in distilled water and sequentially partitioned with different immiscible solvents (petroleum ether, chloroform and ethyl acetate solvents) starting with petroleum ether followed by fractionation with chloroform, and finally ethyl acetate. Ethyl acetate fraction (7 g) was obtained and used for further biological investigation.

### Chemicals

HR-UPLC/MS/MS: Milli-Q water and solvents; formic acid and acetonitrile of LC-MS grade, J. T. Baker (The Netherlands). Nitric oxide and all chemicals used in biological investigation were supplied by Sigma Aldrich Chemie GmbH, St. Louis, MO.

### HR-UPLC-MS/MS analysis

Dried methanol extract (10 mg) was extracted by adding 2 mL of 100% MeOH, containing 10 µgmL^−1^ umbelliferone as an internal standard and sonicated for 20 min with frequent shaking, then centrifuged at 12 000 × g for 10 min to remove debris. A sample of 3 µl of 100% methanol extract was subjected to chromatographic separation using an I-Class UPLC system (Waters Corporation, Milford, USA). The filtered extract through a 0.22-µm filter was subjected to solid-phase extraction using a C18 cartridge (Sep Pack, Waters, Milford, MA, USA) as previously described^11^. UPLC-ESI-qTOF-MS analysis was carried out using an ACQUITY UPLC system (Waters, Milford, MA, USA). Reversed-phase sorbent column: HSS T3 (C_18_, 100 × 1.0 mm), particle size: 1.8 μm: **(**Waters). The annotation of metabolites was based on full mass spectra, molecular formula with an (error < 5 ppm), and by comparing fragmentation pattern with available literature and phytochemical dictionary of natural products database^63^. Chromatographic separation was carried out at 40 °C, using a Waters HSS T3 column (1.0 mm × 100 mm, 1.8 μm) with mobile phases A (0.1% formic acid in water) and B (acetonitrile). The flow rate was set at 0.15 mL/min. The gradient profile was as follows: 0–1 min, 5–5% B; 1–11 min, 5–100% B; 11–19 min, 100% B; 19–20 min, 100%−5% B; 20–25 min, 5% B. Mass spectrometric detection was carried out on Waters Synapt XS mass spectrometer (Waters Corporation, Milford, USA) equipped with an ESI source. The full scan data were acquired from 50 to 1200 Da, using a capillary voltage of 4.0 kV for positive ion mode and 3.0 kV for negative ion mode, sampling cone voltage of 30 V for positive ion mode and 35 V for negative ion mode, extraction cone voltage of 4.0 V, source temperature of 140 °C, cone gas flow of 50 L/h, desolvation gas (N^2^) flow of 1000 L/h and desolvation gas temperature of 450 °C. The collision voltage was set as 5.0 eV for low-energy scan and 25–50 eV for high-energy scan. The collision energy settings (5.0 eV for low-energy scan and 25–50 eV for high-energy scan)^11^ were selected based on instrument manufacturer recommendations and prior optimization studies to ensure effective fragmentation of a wide range of metabolites with diverse structural properties. These values provide a balance between low-energy precursor ion detection and adequate high-energy fragmentation required for structural elucidation in data-independent acquisition (DIA) mode.

### Nitric oxide (NO) Inhibition activity

NO inhibition activity of the tested sample was determined by using a sodium nitroprusside (SNP)^64,65^. NO radical generated from SNP in aqueous solution at physiological pH reacts with oxygen to produce nitrite ions that were measured by the Greiss reagent. The reaction mixture (2 mL) containing various concentrations of the tested samples and SNP (10 mM) in phosphate buffered saline (PBS; pH 7.4) was incubated at 25 ºC for 150 min. At the end of the incubation period, 1 mL of reaction mixture samples was diluted with 1 mL Greiss reagent (1% sulphanilamide (w/v) in 5% phosphoric acid (v/v) and 0.1% naphthyl ethylene diamine dihydrochloride). The mixture was incubated at 25 °C for a further 30 min. The absorbance of these solutions was measured at 546 nm against the corresponding blank solution (without sodium nitroprusside). Resveratrol was used as a reference standard. All the tests were performed in triplicate. The percentage inhibition activity was calculated using the formula:

$$\:\text{I}\text{n}\text{h}\text{i}\text{b}\text{i}\text{t}\text{i}\text{o}\text{n}\:\text{\%}\:=\left[\frac{\text{A}\:\text{c}\text{o}\text{n}\text{t}\text{r}\text{o}\text{l}-\text{A}\:\text{s}\text{a}\text{m}\text{p}\text{l}\text{e}}{\text{A}\:\text{c}\text{o}\text{n}\text{t}\text{r}\text{o}\text{l}}\right]\times 100$$

Where, A _control_ is the absorbance of the control reaction at 546 nm and A_test_ represents the absorbance of a test reaction at the same wavelength. Tested material concentration providing 50% inhibition (IC_50_) was calculated from the graph plotting inhibition percentage against concentration.

### Statistical analysis

The results of biological investigation were analyzed in triplicate and displayed as average ± standard deviation of the mean (SD) (Table S1).

## Electronic supplementary material

Below is the link to the electronic supplementary material.

Supplementary Material 1

## Data Availability

All data generated or analysed during this study are included in this published article [and its supplementary information files].
