# Supplementary material for: UPLC-HRMS-MS profiling of Ludwigia adscendens subsp. diffusa aerial parts and investigation of the anti-inflammatory effect
Source: Sci Rep. 2025 Jun 5;15:19718. doi: 10.1038/s41598-025-05183-x (PMC12137884; doi:10.1038/s41598-025-05183-x)

**UPLC-MS profiling of *Ludwigia adscendens* subsp. *diffusa* aerial parts and investigation of the anti-inflammatory effect**

**Enas M. Shawky ^1^, Rim Hamdy^2^, Mohamed R. Elgindi ^3^, Mostafa H. Baky ^1*^**

*^1^ Department of Pharmacognosy, Faculty of Pharmacy, Egyptian Russian University, Badr City, 11829, Cairo, Egypt*

*^2^* *Department of Botany and Microbiology, Faculty of Science, Cairo University*

*^3^ Department of Pharmacognosy, Faculty of Pharmacy, Helwan University, Cairo, Egypt*

*Corresponding author:

**Mostafa H. Baky**

*Pharmacognosy Department, College of Pharmacy, Egyptian Russian University, Badr City 11829, Cairo, Egypt.*
E-mail addresses: [dr_mostafa1984@yahoo.com](mailto:dr_mostafa1984@yahoo.com), [mostafa-hasan@eru.edu.eg](mailto:mostafa-hasan@eru.edu.eg)

| **Table S1.** Anti-inflammatory activity by NO inhibition of *L. adscendens* aerial parts methanol extract and ethyl acetate extract | | | | |
| --- | --- | --- | --- | --- |
| **Sample conc. (µg/ml)** | **Methanol extract NO inhibition (%)** | **SD** | **Ethyl acetate fraction NO inhibition (%)** | **SD** |
| **0** | **0** | 0 | **0** |  |
| **0.5** | **2.75** | 0.09 | **2.7** | 0.09 |
| **1** | **4.03** | 0.14 | **3.92** | 0.13 |
| **2** | **6.71** | 0.36 | **6.51** | 0.35 |
| **3.9** | **10.62** | 0.61 | **10.24** | 0.61 |
| **7.8** | **30.64** | 1.14 | **29.93** | 1.12 |
| **15.6** | **47.21** | 1.46 | **45.35** | 1.42 |
| **31.25** | **59.08** | 2.15 | **65.32** | 2.1 |
| **62.5** | **68.42** | 2.3 | **69.69** | 2.35 |
| **125** | **76.13** | 2.35 | **78.34** | 2.37 |
| **250** | **83.84** | 2.6 | **88.12** | 2.61 |
| **500** | **91.55** | 2.67 | **91.01** | 2.69 |
| **1000** | **93.56** | 2.69 | **92.65** | 2.69 |


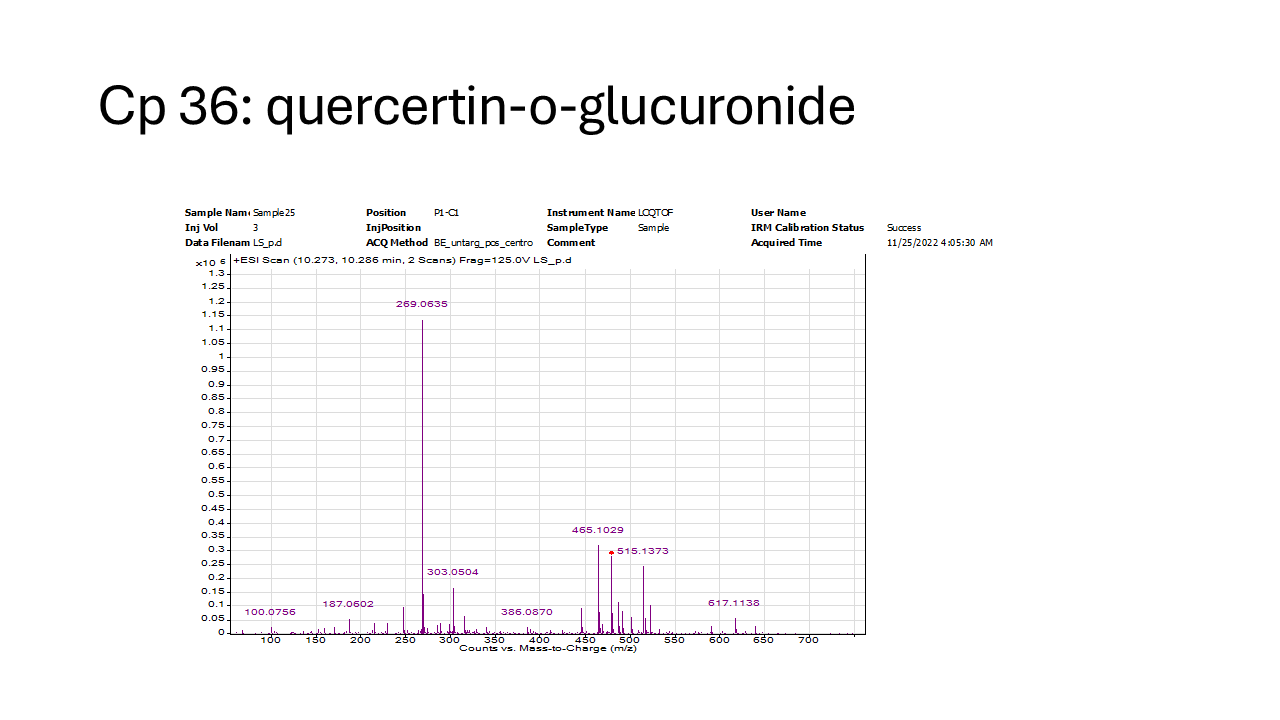

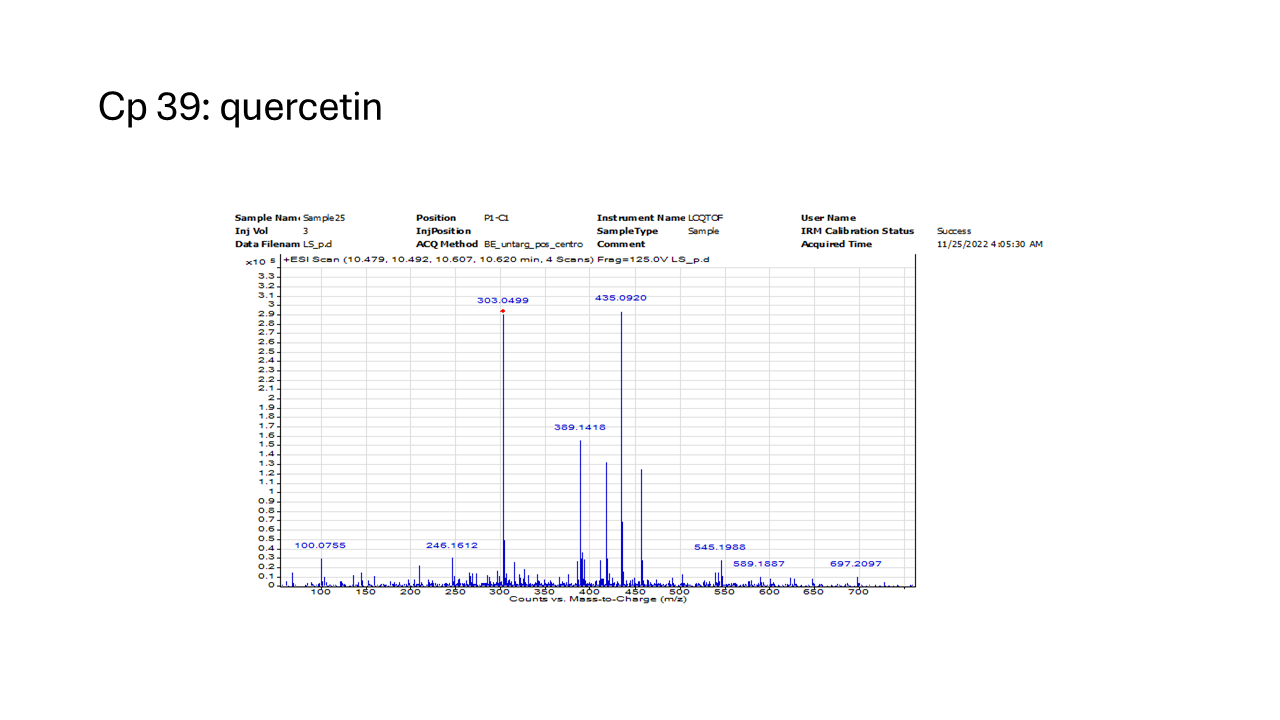

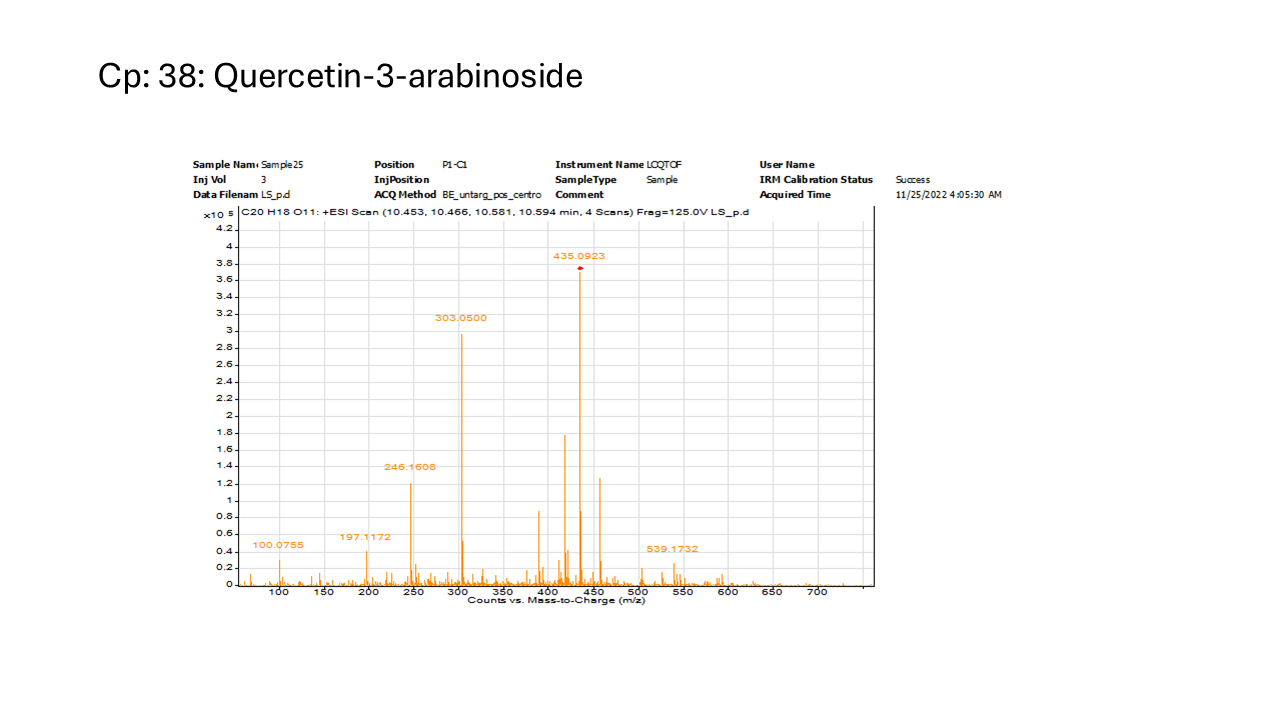

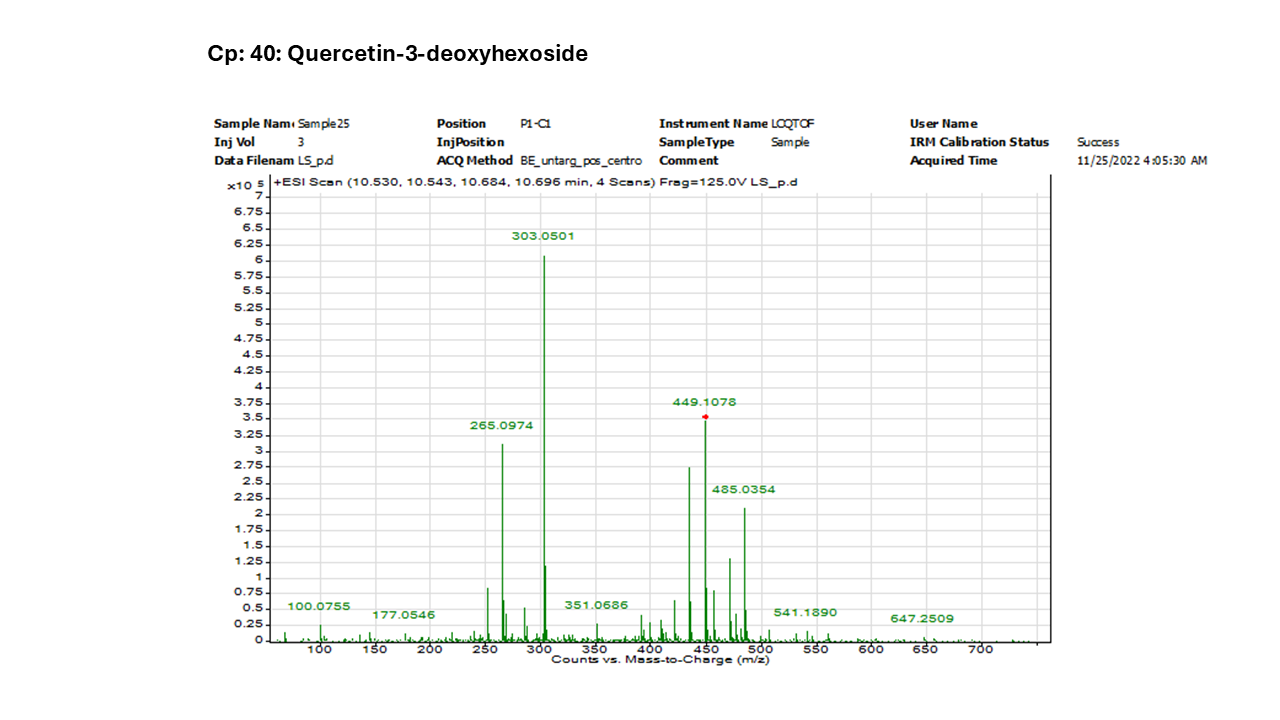

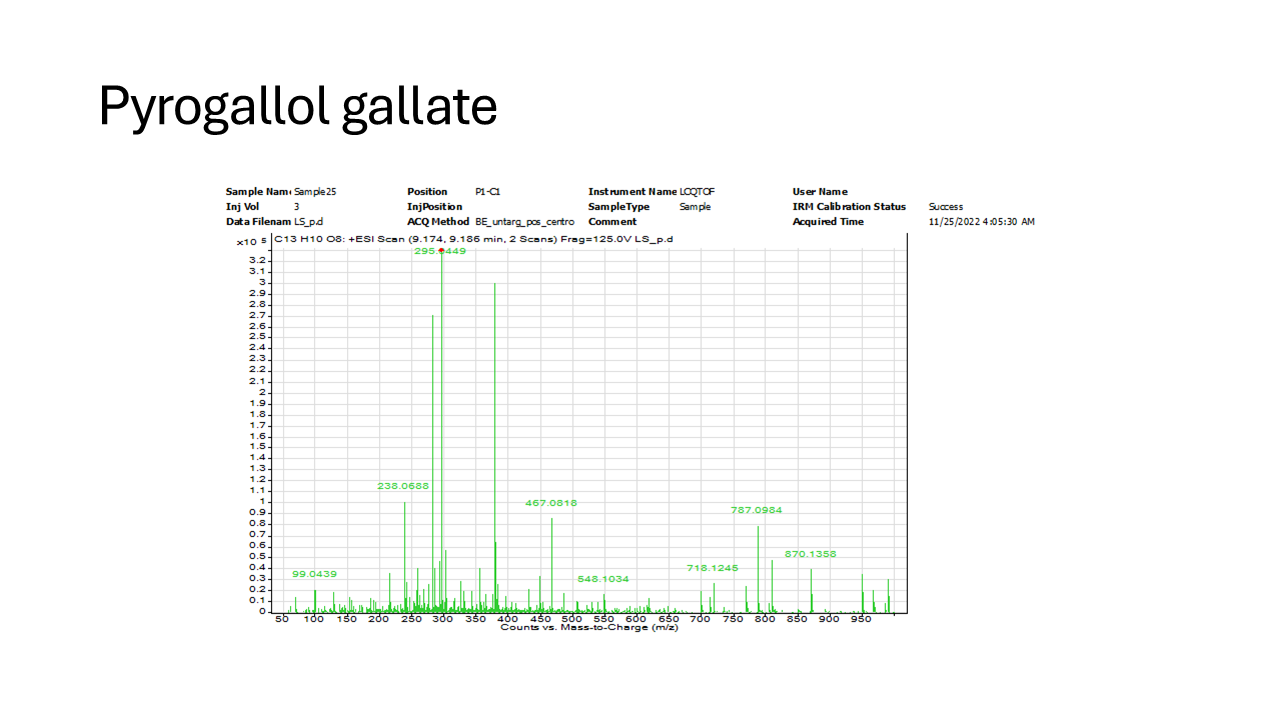

Supplement: Supplementary file 1 — Supplementary Material 1 [file 41598_2025_5183_MOESM1_ESM.docx]
